# Editorial: Next generation MSC therapy manufacturing, potency and mechanism of action analysis

**DOI:** 10.3389/fimmu.2023.1192636

**Published:** 2023-04-21

**Authors:** Raghavan Chinnadurai, Sowmya Viswanathan, Guido Moll

**Affiliations:** ^1^ Department of Biomedical Sciences, Mercer University School of Medicine, Savannah, GA, United States; ^2^ Osteoarthritis Research Program, Division of Orthopedic Surgery, Schroeder Arthritis Institute, University Health Network, Toronto, ON, Canada; ^3^ Krembil Research Institute, University Health Network, Toronto, ON, Canada; ^4^ Institute of Biomedical Engineering, Division of Hematology, Department of Medicine, University of Toronto, Toronto, ON, Canada; ^5^ BIH Center for Regenerative Therapies (BCRT), Berlin, Germany; ^6^ Berlin-Brandenburg School for Regenerative Therapies (BSRT), Berlin, Germany; ^7^ Department of Nephrology and Internal Intensive Care Medicine, all Charité Universitätsmedizin Berlin, corporate member of Freie Universität Berlin, Humboldt-Universität zu Berlin, Berlin Institute of Health (BIH), Berlin, Germany

**Keywords:** mesenchymal stromal/stem cells (MSC), cell product manufacturing, mechanism of action (MOA), safety and efficacy, potency analysis, cell therapy, immunomodulation, and regeneration

## Introduction

**Figure 1:**
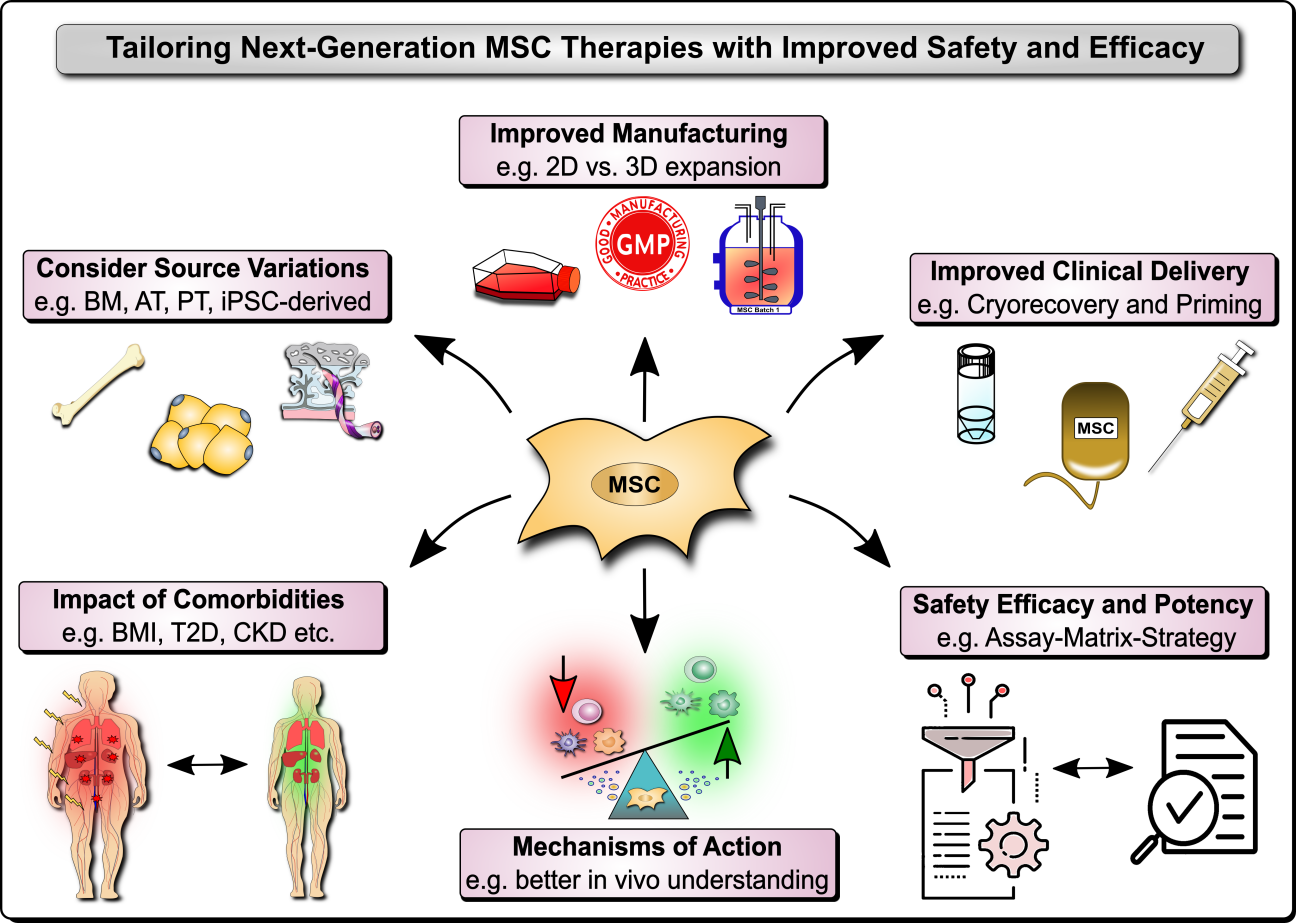
Next Generation MSC Therapy Manufacturing, MoA and Potency Analysis. Next-generation MSC therapy improvements to safety and efficacy include among others: 1) Considerations on variations in the MSC source material, e.g. typically including bone marrow (BM), adipose tissue (AT), perinatal tissue (PT), and induced pluripotent stem cell (iPSC)-derived MSC products; 2) The relevant impact of donor comorbidities, e.g. the role of the body mass index (BMI), obesity, and the typically associated common comorbidities that are increasing in the population, such as type 2 diabetes (T2D) and chronic kidney disease (CKD); 3) Improved MSC product manufacturing, e.g. 2D vs. 3D expansion and in particular the anticipation of the degree of cell expansion and respective loss of potency, but also concomitant safety considerations; 4) Improved clinical delivery of MSC products, e.g. anticipating the role of cryopreservation and freeze-thawing, but also various cell priming strategies, such as cytokine and mechano-transduction licensing; and 5) Better understanding of the mechanisms of action (MoA) of MSC products *in vitro* and particularly *in vivo* in respective patients and clinical cohorts with their very own specific requirements and covariates that may confound treatment safety and efficacy; and 6) Coordinated and relevant safety, efficacy, and potency Assessment with suitable approaches, e.g. the combinatorial assay-matrix-approach, and concomitant potency screening but also potency and safety improvements.

Mesenchymal stromal/stem cells (MSCs) are non-hematopoietic cells found in vascularized tissues and organs, that possess profound immunomodulatory and regenerative properties, which warrant their application in cellular and regenerative therapy (1–7). Regulatory authorities have already approved MSC therapies for several clinical conditions, such as Graft-versus-Host Disease (GvHD), Perianal Fistula in Crohn’s Disease, and Critical Limb Ischemia (6, 7). However, there are still some limitations with this novel type of cell therapy that need to be understood and addressed, and thus form the basis for this and other earlier Research Topics (2, 3). These concerns are mainly due to contradictory results on MSCs’ therapeutic efficacy profile in preclinical models compared to real-world experience in different clinical indications (7–9). In addition, there are also some minor safety concerns related to systemic infusion that should not be overlooked (4, 5, 10). However, both efficacy and safety limitations may be overcome through improved understanding of MSC product properties, handling, and function (2, 4, 5, 9, 11–19). Indeed, any remaining limitations with this novel type of therapy may be largely due to variations in MSC products, their manufacturing practices, a lack of understanding on their optimal clinical delivery, their *in vivo* mechanism of action (MoA), and the concomitant clinical indications, and in particular the insufficient clinical potency assessment (4, 5, 9, 11–23). In this Research Topic, we have collected 13 original research and review articles that address new strategies for improved manufacturing, and MoA and potency assessment in clinical trials, for the design of next-generation MSC therapies with optimal clinical efficacy and safety **Figure 1**.

## Fresh vs frozen thawed MSCs

Clinical use of cryopreserved “off-the-shelf” MSC products is a feasible strategy in which cryopreserved cells are thawed near the bedside and infused immediately into the patients (11). This strategy has been discussed as a potential confounder of MSC efficacy since preclinical data have shown a discrepant functionality of MSCs immediately post thawing (11, 16, 24–31). These prompted insights on the clinical testing of “fresh MSCs” either derived from actively growing culture or post thaw culture rescued from cryopreservation. Stenger et al. tested the safety of autologous “fresh MSCs” in 11 patients with GvHD (n=4 Acute; n=7 Chronic). Culture rescue was deployed in a multi-dosing strategy where bone marrow (BM)-MSCs were expanded and cryobanked from a single BM aspiration. Subsequently, the cells were thawed and culture rescued for 72 hours prior to infusion. Intravenous (IV) infusions of fresh MSCs were well tolerated in these patients and no dose associated toxicity was observed. Three out of four acute GvHD patients displayed partial to complete responses to fresh MSCs. In chronic GvHD, three-month overall responses were partial (n=5), stable (n=1) and progressive (n=1). Although this study’s primary endpoint is safety, the efficacy data, particularly for acute GvHD, are encouraging, since the responses are equivalent to FDA approved second line treatments for steroid resistant GvHD. Ekpo et al. put forth an opinion paper on cryopreservation of cell-based drug delivery systems. The authors emphasize, when stem cells (ex. MSCs) are utilized as a vehicle for drug delivery, cryopreservation formulations need to be well researched, since the cryopreservation process could negatively impact the functionality of drug formulations and their therapy efficacy. Fernandez-Santos et al. provided a general guide, including rules and legislation, for homogenous MSC manufacturing, cell banks, optimal cryopreservation and post thaw potency assessments for improved therapy. In support, Willer et al. demonstrated that pooled human BM-MSCs during cell manufacturing process minimize product variations and accelerate effective wound healing in the animal model. This “decision-making approach” identified the preferable use of fresh MSCs over readily thawed MSCs and the significance of repeated delivery for future clinical wound healing studies. Accomplishment of sustained efficacy of MSCs is imminent in moving forward and thus inclusion of the “Freeze-Thawing” confounder in the cell manufacturing needs to be taken into consideration.

## Potency metrics of MSCs

Potency analysis is now often mandatory for determining MSCs’ release criteria as cellular therapeutics in advanced clinical trials and marketing approval depending on the regional regulatory requirements (32). Considering the complex MoA and the involvement of more than one single effector molecule/pathway associated with MSCs’ functionality, a “combinatorial-potency-assay-matrix” approach to define MSC potency has recently been proposed by the International Society for Cell Therapy (ISCT) (22, 30). Robb et al. developed an innovative, sensitive and quantitative assay matrix strategy to define putative critical quality attributes of adipose tissue (AT)-derived AT-MSCs and to distinguish some critical processing parameters and the impact of donor heterogeneity. This strategy included combinatorial analysis of AT-MSCs’ morphometrics, gene and protein multiplex, and functionality, such as macrophage polarization and angiogenic fitness. This multivariate assay-matrix-strategy identified panels of putative critical quality attributes for immunomodulatory and angiogenesis fitness (with minimum and maximum value ranges), which can be used to screen culture conditions and potential donors for optimal MSC potency. Wiese et al. deployed a robust and standardized potency assay to identify tissue specific effector molecules on MSCs. Umbilical cord (UC)- and BM-derived MSCs were compared with and without exogenous cytokine activation for the enumeration and quantification of effector genes and soluble analytes as a surrogate measure of potency, to correlate them in the future with functional clinical outcomes (positive/negative). This cytokine activation strategy attenuated heterogeneity of unstimulated MSC populations and thus can inform a more standardized potency assay.

## Augmentation of MSC’s potency

First generation clinical trials largely employed MSCs in their non-activated “resting stage”, while preclinical studies provided pathway to inform on second generation clinical trials with augmented potency involving not only primed/activated/preconditioned MSCs (33), but also their products such as exosomes and extracellular vesicles (EVs) (34). Hackel et al. compared the immunoregulatory properties of unstimulated and cytokine-cocktail-licensed/primed MSCs and their EVs, to obtain more robust therapeutic responses *in vivo*. EVs derived from cytokine-cocktail-primed MSCs displayed enhanced therapeutic efficacy in the animal model of GvHD, which was abrogated with the blockade of PD1-PDL1/PDL2 pathway. This strategy provided insights that EVs from primed MSCs can be used therapeutically with augmented potency. In contrast to cytokine mediated priming, Skibber et al. deployed a mechano-transduction strategy with distinct biomechanical cue named Wall Shear Stress (WSS) to enhance MSC potency. The WSS-conditioning did not affect MSCs’ viability and identity, but enhanced their immunomodulatory potency. This mechanotransduction mediated priming is an exciting step forward, since it can be easily translated to enhance MSCs’ potency. Boland et al. reviewed the challenges of employing resting MSCs in patients with comorbidities, such as obesity, since obese microenvironment alters the immunomodulatory functions of MSCs (35). The authors propose that “one size fits all” strategy may not work when considering diverse comorbidities. Utilizing such an approach may not only mitigate the potency of MSCs, but also compromise patient safety due to the thromboembolic nature of obesity and its associated cardiovascular comorbidities (2, 4–6, 10, 14, 15, 19, 36, 37). Consistent with other studies, the authors propose that clinical studies should consider priming of MSCs and anti-thrombotic prophylaxis for patients with obesity and metabolic disorders, to lower any apparent risk of severe thromboembolic events (e.g. venous or pulmonary thromboembolism), which is a well-known potential side-effect of MSC infusion undertaken without the necessary precautions or awareness (2, 4–6, 10, 13–15, 19, 36, 37).

## Reprogramming and genetic manipulation of MSCs

Although MSCs are considered more-than-minimally-manipulated cell therapy products by regulatory authorities, advances are necessary to reprogram and genetically manipulate MSCs for the management of certain illness. Balina-Sanchez et al. demonstrated the feasibility of reprogramming and generating induced pluripotent stem cell (iPSC)-derived MSCs from urine epithelial cells of pediatric patients with brain tumor. This study also showed that these reprogrammed MSC populations from brain tumor patients are equivalent to healthy controls in their immunomodulatory functions. This brings insights on the utility of non-invasive technology to manufacture MSCs for the investigational clinical use in pediatric patients. Ramamurthy et al. detailed challenges and drawbacks of gene editing/addition strategies to produce FVIII in placenta derived MSCs. Although reporter genes can be efficiently inserted to the specific locus utilizing CRISPR/CAS9 strategy, transgene of FVIII could not be knocked in due to the size limitation. Transgene or CRISPR/CAS9 introduction using plasmids upregulates several proinflammatory Toll Like Receptors and stress responses in endoplasmic reticulum which can intervene MSCs’ functionality. These raise caution when utilizing gene addition strategies on human MSCs.

## MSC therapy for COVID 19 and mechanism of action

MSC’s beneficial lung homing, immunosuppressive and regenerative properties have attracted their use to mitigate acute respiratory distress syndrome (ARDS) resulting from coronavirus-induced disease-2019 (COVID-19) (6, 10, 38). Gregoire et al. tested the safety and efficacy of IV infusions of BM-MSCs in eight patients with severe COVID-19 who were admitted in intensive care unit. No adverse effect related to MSC infusion was observed in these patients. Retrospect comparison with the matched patient controls has demonstrated that survival is significantly higher for patients receiving MSC therapy. In contrast to Stenger et al., this clinical trial utilized immediately thawed MSCs, which is a feasible strategy in medical emergency management situations. However, preclinical data also has demonstrated that cytokine priming strategies and other cryopreservation optimization strategies can be deployed to attenuate the cellular injury associated with freeze thawing (11, 16, 27, 31, 39, 40). Adoption of these strategies in cell manufacturing and clinical utilization would further enhance the efficacy of MSCs for clinical emergency management. Nevertheless, the MoA of MSCs in executing anti-inflammation and immunoregulation in mitigating the severity of COVID-19 akin to ARDS is yet to be understood (2, 10, 41). Indeed, the MoA of MSCs upon infusion into patients is highly complex, and this knowledge is still developing (5, 10, 41), though at least three major MoAs has been proposed including differentiation into mesodermal tissues, modulation of immune cells, and in particular the polarization of macrophages with efferocytosis of apoptotic MSCs (23, 42, 43), although *in vivo* engraftment and differentiation of MSCs is only transient and very minimal at least in part due to triggering of the Instant Blood-Mediated Inflammatory Reaction (IBMIR) and the concomitant rapid destruction of the majority of the infused cells (4, 5, 12–16, 27, 39); Indeed, typically >80% of the therapeutic cells are lost within the first 24 hours post infusion. Zheng et al. provided key insights on the MoA of MSCs involving efferocytosis, a phenomenon in which apoptotic debris is cleared by phagocytes and maintain or restore homeostasis (8, 31, 43). They discussed the role of resident and migratory phagocytic cells of the secondary lymphoid organs in mediating MSCs’ therapeutic effect. The role of efferocytosis and associated phagocytes in the secondary lymphoid organs in mediating MSCs’ therapeutic effect in COVID needs further investigation.

## Conclusion

The horizon for the use of next generation engineered MSCs appears bright with both genetic and non-genetic engineering strategies emerging. Together with quantitative approaches to fully and carefully characterize MSC potency attributes, the editors of this series are optimistic that the next generation MSCs will be more efficacious in clinical trial outcomes and bridge the gap to clinical and commercial success.

## Author contributions

RC drafted the first version. GM drafted the figure. RC, SV and GM have made a substantial, direct, and intellectual contribution to the writing and approved it for publication. All authors listed have made a substantial, direct, and intellectual contribution to the work and approved it for publication.

## References

[1] Viswanathan S Shi Y Galipeau J Krampera M Leblanc K Martin I. Mesenchymal stem versus stromal cells: international society for cell & gene therapy (ISCT^®^) mesenchymal stromal cell committee position statement on nomenclature. Cytotherapy (2019) 21:1019–24. doi: 10.1016/j.jcyt.2019.08.002 31526643

[2] Moll G Hoogduijn MJ Ankrum JA. Editorial: Safety, efficacy and mechanisms of action of mesenchymal stem cell therapies. Front Immunol (2020) 11:243. doi: 10.3389/fimmu.2020.00243 32133010 PMC7040069

[3] Capilla-González V Herranz-Pérez V Sarabia-Estrada R Kadri N Moll G. Editorial: mesenchymal stromal cell therapy for regenerative medicine. Front Cell Neurosci (2022) 16. 10.3389/fncel.2022.932281 PMC9179645 35693887

[4] Moll G Ankrum JA Kamhieh-Milz J Bieback K Ringdén O Volk HD. Intravascular mesenchymal Stromal/Stem cell therapy product diversification: time for new clinical guidelines. Trends Mol Med (2019). doi: 10.1016/j.molmed.2018.12.006 30711482

[5] Moll G Ankrum JA Olson SD Nolta JA. Improved MSC minimal criteria to maximize patient safety: a call to embrace tissue factor and hemocompatibility assessment of MSC products. Stem Cells Trans Med (2022) 11:2–13. doi: 10.1093/stcltm/szab005 PMC8895495 35641163

[6] Ringdén O Moll G Gustafsson B Sadeghi B. Mesenchymal stromal cells for enhancing hematopoietic engraftment and treatment of graft-versus-Host disease, hemorrhages and acute respiratory distress syndrome. Front Immunol (2022). doi: 10.3389/fimmu.2022.839844 PMC8973075 35371003

[7] Levy O Kuai R Siren EMJ Bhere D Milton Y Nissar N. Shattering barriers toward clinically meaningful MSC therapies. Sci Adv (2020) 6:eaba6884. doi: 10.1126/sciadv.aba6884 32832666 PMC7439491

[8] Galipeau J Sensebe L. Mesenchymal stromal cells: clinical challenges and therapeutic opportunities. Cell Stem Cell (2018) 22:824–33. doi: 10.1016/j.stem.2018.05.004 PMC6434696 29859173

[9] Robb KP Fitzgerald JC Barry F Viswanathan S. Mesenchymal stromal cell therapy: progress in manufacturing and assessments of potency. Cytotherapy (2019) 21:289–306. doi: 10.1016/j.jcyt.2018.10.014 30528726

[10] Moll G Drzeniek N Kamhieh-Milz J Geissler S Volk H-D Reinke P. MSC therapies for COVID-19: Importance of patient coagulopathy, thromboprophylaxis, cell product quality and mode of delivery for treatment safety and efficacy. Front Immunol (2020) 11:1091. doi: 10.3389/fimmu.2020.01091 32574263 PMC7249852

[11] Cottle C Porter AP Lipat A Turner-Lyles C Nguyen J Moll G. Impact of cryopreservation and freeze-thawing on therapeutic properties of mesenchymal Stromal/Stem cells and other common cellular therapeutics. Curr Stem Cell Rep (2022) 8:72–92. doi: 10.1007/s40778-022-00212-1 35502223 PMC9045030

[12] Moll G Jitschin R von Bahr L Rasmusson-Duprez I Sundberg B Lonnies L. Mesenchymal stromal cells engage complement and complement receptor bearing innate effector cells to modulate immune responses. PLoS One (2011) 6:e21703. doi: 10.1371/journal.pone.0021703 21747949 PMC3128611

[13] Von Bahr L Batsis I Moll G Hägg M Szakos A Sundberg B. Analysis of tissues following mesenchymal stromal cell therapy in humans indicates limited long-term engraftment and no ectopic tissue formation. Stem Cells (2012) 30:1575–8. doi: 10.1002/stem.1118 22553154

[14] Moll G Rasmusson-Duprez I von Bahr L Connolly-Andersen AM Elgue G Funke L. Are therapeutic human mesenchymal stromal cells compatible with human blood? Stem Cells (2012) 30:1565–74. doi: 10.1002/stem.1111 22522999

[15] Moll G Ignatowicz L Catar R Luecht C Sadeghi B Hamad O. Different procoagulant activity of therapeutic mesenchymal stromal cells derived from bone marrow and placental decidua. Stem Cells Dev (2015) 24:2269–79. doi: 10.1089/scd.2015.0120 26192403

[16] Moll G Le Blanc K. Engineering more efficient multipotent mesenchymal stromal (stem) cells for systemic delivery as cellular therapy. ISBT Sci Ser (2015) 10:357–65. doi: 10.1111/voxs.12133

[17] Nolta JA Galipeau J Phinney DG. Improving mesenchymal stem/stromal cell potency and survival: proceedings from the international society of cell therapy (ISCT) MSC preconference held in may 2018, palais des congres de Montreal, organized by the ISCT MSC scientific committee. Cytotherapy (2020) 22:123–6. doi: 10.1016/j.jcyt.2020.01.004 32067856

[18] Galipeau J Krampera M Leblanc K Nolta JA Phinney DG Shi Y. Mesenchymal stromal cell variables influencing clinical potency: the impact of viability, fitness, route of administration and host predisposition. Cytotherapy (2021). doi: 10.1016/j.jcyt.2020.11.007 PMC11708105 33714704

[19] Caplan H Olson SD Kumar A George M Prabhakara KS Wenzel P. Mesenchymal stromal cell therapeutic delivery: Translational challenges to clinical application. Front Immunol (2019) 10:1645. doi: 10.3389/fimmu.2019.01645 PMC6685059 31417542

[20] Garcia-Bernal D Garcia-Arranz M Yanez RM Hervas-Salcedo R Cortes A Fernandez-Garcia M. The current status of mesenchymal stromal cells: controversies, unresolved issues and some promising solutions to improve their therapeutic efficacy. Front Cell Dev Biol (2021) 9:650664. doi: 10.3389/fcell.2021.650664 33796536 PMC8007911

[21] Chinnadurai R Rajakumar A Schneider AJ Bushman WA Hematti P Galipeau J. Potency analysis of mesenchymal stromal cells using a phospho-STAT matrix loop analytical approach. Stem Cells (2019) 37:1119–25. doi: 10.1002/stem.3035 PMC6729138 31108008

[22] Galipeau J Krampera M Barrett J Dazzi F Deans RJ Debruijn J. International society for cellular therapy perspective on immune functional assays for mesenchymal stromal cells as potency release criterion for advanced phase clinical trials. Cytotherapy (2016) 18:151–9. doi: 10.1016/j.jcyt.2015.11.008 PMC4745114 26724220

[23] Krampera M Le Blanc K. Mesenchymal stromal cells: putative microenvironmental modulators become cell therapy. Cell Stem Cell (2021) 28:1708–25. doi: 10.1016/j.stem.2021.09.006 34624232

[24] Galipeau J. The mesenchymal stromal cells dilemma–does a negative phase III trial of random donor mesenchymal stromal cells in steroid-resistant graft-versus-host disease represent a death knell or a bump in the road? Cytotherapy (2013) 15:2–8. doi: 10.1016/j.jcyt.2012.10.002 23260081

[25] Galipeau J. Concerns arising from MSC retrieval from cryostorage and effect on immune suppressive function and pharmaceutical usage in clinical trials. ISBT Sci Ser (2013) 8:100–1. doi: 10.1111/voxs.12022

[26] Francois M Copland IB Yuan S Romieu-Mourez R Waller EK Galipeau J. Cryopreserved mesenchymal stromal cells display impaired immunosuppressive properties as a result of heat-shock response and impaired interferon-gamma licensing. Cytotherapy (2012) 14:147–52. doi: 10.3109/14653249.2011.623691 PMC3279133 22029655

[27] Moll G Alm JJ Davies LC von Bahr L Heldring N Stenbeck-Funke L. Do cryopreserved mesenchymal stromal cells display impaired immunomodulatory and therapeutic properties? Stem Cells (2014) 32:2430–42. doi: 10.1002/stem.1729 PMC4381870 24805247

[28] Chinnadurai R Garcia MA Sakurai Y Lam WA Kirk AD Galipeau J. Actin cytoskeletal disruption following cryopreservation alters the biodistribution of human mesenchymal stromal cells. vivo Stem Cell Rep (2014) 3:60–72. doi: 10.1016/j.stemcr.2014.05.003 PMC4110775 25068122

[29] Chinnadurai R Copland IB Garcia MA Petersen CT Lewis CN Waller EK. Cryopreserved mesenchymal stromal cells are susceptible to T-cell mediated apoptosis which is partly rescued by IFNgamma licensing. Stem Cells (2016) 34:2429–42. doi: 10.1002/stem.2415 PMC5016228 27299362

[30] Chinnadurai R Rajan D Qayed M Arafat D Garcia M Liu YF. Potency analysis of mesenchymal stromal cells using a combinatorial assay matrix approach. Cell Rep (2018) 22:2504–17. doi: 10.1016/j.celrep.2018.02.013 PMC5855117 29490284

[31] Moll G Geissler S Catar R Ignatowicz L Hoogduijn MJ Strunk D. Cryopreserved or fresh mesenchymal stromal cells: only a matter of taste or key to unleash the full clinical potential of MSC therapy? Adv Exp Med Biol (2016) 951:77–98. doi: 10.1007/978-3-319-45457-3_7 27837556

[32] Goldsobel G von Herrath C Schlickeiser S Brindle N Stähler F Reinke P. RESTORE survey on the public perception of advanced therapies and ATMPs in Europe-why the European union should invest more! Front Med (Lausanne) (2021) 8:739987. doi: 10.3389/fmed.2021.739987 34765617 PMC8576137

[33] Doorn J Moll G Le Blanc K van Blitterswijk C de Boer J. Therapeutic applications of mesenchymal stromal cells: paracrine effects and potential improvements. Tissue engineering Part B Rev (2012) 18:101–15. doi: 10.1089/ten.TEB.2011.0488 21995703

[34] Lener T Gimona M Aigner L Borger V Buzas E Camussi G. Applying extracellular vesicles based therapeutics in clinical trials - an ISEV position paper. J Extracell Vesicles (2015) 4:30087. doi: 10.3402/jev.v4.30087 26725829 PMC4698466

[35] Soria-Juan B Escacena N Capilla-González V Aguilera Y Llanos L Tejedo JR. Cost-effective, safe, and personalized cell therapy for critical limb ischemia in type 2 diabetes mellitus. Olson SD, Kumar A, George M, Prabhakara KS, Wenzel P, (2019) 10:1151. doi: 10.3389/fimmu.2019.01151 PMC6558400 31231366

[36] Giri J Moll G. MSCs in space: mesenchymal stromal cell therapeutics as enabling technology for long-distance manned space travel. Curr Stem Cell Rep (2022) 8:1–13.

[37] Wu Z Zhang S Zhou L Cai J Tan J Gao X. Thromboembolism induced by umbilical cord mesenchymal stem cell infusion: a report of two cases and literature review. Transplant Proc (2017) 49:1656–8. doi: 10.1016/j.transproceed.2017.03.078 28838459

[38] Kirkham AM Bailey AJM Shorr R Lalu MM Fergusson DA Allan DS. Systematic review and meta-analysis of randomized controlled trials of mesenchymal stromal cells to treat coronavirus disease 2019: is it too late? Cytotherapy (2023) 25:341–52. doi: 10.1016/j.jcyt.2022.10.00 PMC9556962 36333234

[39] Moll G Hult A von Bahr L Alm JJ Heldring N Hamad OA. Do ABO blood group antigens hamper the therapeutic efficacy of mesenchymal stromal cells? PLoS One (2014) 9:e85040. doi: 10.1371/journal.pone.0085040 24454787 PMC3890285

[40] Hoogduijn MJ de Witte SF Luk F van den Hout-van Vroonhoven MC Ignatowicz L Catar R. Effects of freeze-thawing and intravenous infusion on mesenchymal stromal cell gene expression. Stem Cells Dev (2016) 25:586–97. doi: 10.1089/scd.2015.032 26914168

[41] Weiss DJ Filiano A Galipeau J Khoury M Krampera M Lalu M. An international society for cell and gene therapy mesenchymal stromal cells committee editorial on overcoming limitations in clinical trials of mesenchymal stromal cell therapy for coronavirus disease-19: time for a global registry. Cytotherapy (2022) 24:1071–3. doi: 10.1016/j.jcyt.2022.07.010 PMC9339970 36028438

[42] Galleu A Riffo-Vasquez Y Trento C Lomas C Dolcetti L Cheung TS. Apoptosis in mesenchymal stromal cells induces *in vivo* recipient-mediated immunomodulation. Sci Trans Med (2017) 9. doi: 10.1126/scitranslmed.aam7828 29141887

[43] Galipeau J. Macrophages at the nexus of mesenchymal stromal cell potency: the emerging role of chemokine cooperativity. Stem Cells (2021). doi: 10.1002/stem.3380 PMC8453730 33786935

